# One-step, high-yield synthesis of g-C_3_N_4_ nanosheets for enhanced visible light photocatalytic activity

**DOI:** 10.1039/c9ra08922e

**Published:** 2019-11-29

**Authors:** Liyan Wang, Yangwen Hou, Shanshan Xiao, Fei Bi, Li Zhao, Yingqi Li, Xiaojia Zhang, Guangqing Gai, Xiangting Dong

**Affiliations:** Key Laboratory of Building Energy-Saving Technology Engineering, College of Materials Science and Engineering, Jilin Jianzhu University Changchun P. R. China gaigq@163.com +86-431-84566095 +86-431-84566095; Key Laboratory of Applied Chemistry and Nanotechnology at Universities of Jilin Province, Changchun University of Science and Technology Changchun P. R. China dongxiangting888@163.com +86-431-85383815 +86-431-85582574

## Abstract

A facile template-free one-step synthesis method of ultrathin g-C_3_N_4_ nanosheets was developed through thermal polycondensation of melamine. The higher temperature, prolonged time and tightly sealed crucible reaction system contributed to the formation of ultrathin g-C_3_N_4_ nanosheets. The as-synthesized g-C_3_N_4_ nanosheets were applied to the visible light photocatalytic degradation of RhB. The photocatalytic activity was significantly enhanced with increased calcination temperature from 500 °C to 650 °C and prolonged calcination time from 4 h to 10 h. Interestingly, the obtained ultrathin g-C_3_N_4_ nanosheets simultaneously possess high yield and excellent photocatalytic activity. Moreover, g-C_3_N_4_ nanosheets can maintain photochemical stability after five consecutive runs. The remarkably enhanced photocatalytic activity can be interpreted as the synergistic effects of the enhanced crystallinity, the large surface area, the reduced layer thickness and size and the reduced number of defects. A new layer exfoliation and splitting mechanism of the formation of the ultrathin nanosheets was proposed. This work provides a new strategy to develop a facile eco-friendly template-free one-step synthesis method for potential large-scale synthesis of ultrathin nanosheets with high yield, high photocatalytic efficiency and stable activity for environmental and energetic applications.

## Abstract 2

g-C_3_N_4_ with enhanced photocatalytic activity and high yield is synthesized by a one-step pyrolysis method of melamine in a sealed crucible.

## Graphical abstract

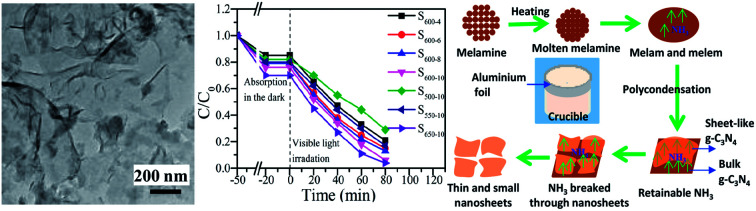

## Introduction

Visible-light photocatalysts have attracted enormous attention worldwide owing to their potential applications in organic pollutant degradation and hydrogen evolution.^1–9^ As a metal-free polymer semiconductor, graphitic carbon nitride (g-C_3_N_4_) is regarded as a promising visible-light photocatalyst due to its reliable chemical and thermal stability.^3–6^ To our knowledge, many strategies have already been developed to improve the activity of g-C_3_N_4,_ such as introducing metallic or nonmetallic elements,^10–14^ construction of a g-C_3_N_4_-based heterojunction^15–17^ and controlling the morphology.^18–21^ The morphological structure of g-C_3_N_4_ significantly affects its performance. In general, g-C_3_N_4_ with favorable nanoarchitecture necessarily exhibits prominent activities in practical applications.^21^ At present, the morphological structure of g-C_3_N_4_ includes bulk, three dimensional (3D),^22,23^ two-dimensional (2D), one-dimensional (1D),^24,25^ and zero-dimensional (0D) nanostructures.^26^ The different morphological structure can be controlled by employing different species and ratios of precursors, condensation temperature, exfoliation and doping methods, and different templating strategies, *et al.*^6^

As is known to all, bulk g-C_3_N_4_ photocatalysts possess low photocatalytic activity due to its small surface area and high recombination rate of photo-generated electron–hole pairs. Since the discovery of graphene, two-dimensional (2D) nanosheets, especially with molecular thickness, have attracted more and more attention in heterogeneous photocatalysts.^27–34^ These nanosheets possess exceptional electronic structures feature of 2D anisotropy with nanometer thickness, resulting in distinctive physicochemical properties owing to the quantum confinement effect (QCE).^35^ Considering that the layer of g-C_3_N_4_ is composed of C–N bonds, and weak van der Waals force exists between the layers, researchers fabricated mono or a few layer C_3_N_4_ sheets by the exfoliation of layered g-C_3_N_4_ to improve its photocatalytic activity.^6^

Recently, considered as an effective pathway to prepare the ultrathin 2D nanosheets of g-C_3_N_4_, the liquid exfoliation technology is developed rapidly. Zhang *et al.* prepared successfully ultrathin g-C_3_N_4_ nanosheets by water exfoliation from bulk g-C_3_N_4_.^27^ Xu *et al.* obtained g-C_3_N_4_ nanosheet with a single atomic thickness of 0.4 nm by a simple chemical exfoliation method.^28^ Lin *et al.* prepared monolayer C_3_N_4_ nanosheet by the pyrolysis of melamine at 550 °C for 4 h, mixed solvent exfoliation and ultrasonic dispersion of 10 h.^29^ Tong *et al.* developed g-C_3_N_4_ nanosheet with high yield *via* a moderate exfoliation method using diluted H_2_SO_4_ suspension of bulk g-C_3_N_4_.^30^ In addition, the thermal exfoliation is considered to be another effective method of preparing the ultrathin 2D nanosheets from bulk g-C_3_N_4_. Dong *et al.* synthesized porous g-C_3_N_4_ nanosheets *via* direct pyrolysis of thiourea followed by a thermal exfoliation.^31^ Niu *et al.* synthesized g-C_3_N_4_ nanosheet with a thickness of around 2 nm by thermal oxidation etching of bulk g-C_3_N_4_ in air.^32^ Qiu *et al.* synthesized g-C_3_N_4_ nanosheet by exfoliating bulk g-C_3_N_4_ with a thermal treatment under H_2_.^33^ Liang *et al.* prepared holey g-C_3_N_4_ nanosheets with abundant in-plane holes by thermally treating bulk g-C_3_N_4_ under an NH_3_ atmosphere.^34^ From the above literature reports, we can draw such a conclusion that the preparation method of ultrathin nanosheets of g-C_3_N_4_ consists of two steps generally. At first, the bulk g-C_3_N_4_ is prepared by thermal polymerization of organic precursors; then ultrathin nanosheets are prepared by the liquid exfoliation and ultrasonic dispersion, or the thermal exfoliation at a certain atmosphere.

In order to simplify the experimental conditions and steps, further realize large-scale production of the ultrathin g-C_3_N_4_ nanosheets, researchers developed one-step synthesis methods by using self-supporting or additive atmosphere as bubble template. For example, Liu *et al.* synthesized g-C_3_N_4_ by the simple thermal pyrolysis of urea without additive assistance, but a typical yield of the powder was only 4 wt%.^36^ Dong *et al.* synthesized porous g-C_3_N_4_ nanosheets by direct pyrolysis of urea by a template-free through prolonging the pyrolysis time.^37^ But the corresponding yield of g-C_3_N_4_ did not be mentioned. As a low-cost and abundant industrial material, urea is regard as an active precursor for preparing porous g-C_3_N_4_. However, this method has a drawback of the low yield, which would limit its practical application. Zhang *et al.* prepared porous g-C_3_N_4_ with yield of 18% by pyrolysis of dicyandiamide precursor using urea as bubble template.^38^ He *et al.* synthesized uniform porous g-C_3_N_4_ through thermal condensation of melamine using sublimed sulfur as soft-template agent, yet the yield was not reported.^39^ Mahalingam *et al.* synthesized g-C_3_N_4_ by one-step pyrolysis reaction of melamine in a semi-sealed alumina crucible, the obtained g-C_3_N_4_ and *n*-Bu_4_N^+^Br^−^ combination was used for epoxide to cyclic carbonate conversion.^40^ Song *et al.* synthesized g-C_3_N_4_ by heating urea at 550 °C for 4 h at a heating of 5 °C min^−1^, UV-visible photocatalytic activity was studied, but visible photocatalytic activity was not studied.^41^

Although variety of strategies have been employed to get g-C_3_N_4_ nanosheets, how to synthesize g-C_3_N_4_ nanosheets with high yield and high photocatalytic activity simultaneously is far from satisfactory. In the present work, we developed a facile template-free one-step synthesis method of ultrathin 2D g-C_3_N_4_ nanosheets through thermal polycondensation of melamine. The formation of 2D g-C_3_N_4_ nanosheet depends mainly on the high calcination temperature, the prolonged calcination time and tightly sealed reaction system. The microstructure, crystal structure and chemical composition, optical property and surface area of as-prepared g-C_3_N_4_ were characterized in detail. The photocatalytic activity of g-C_3_N_4_ nanosheets were systematically investigated by degradation of RhB under the visible light irradiation. Satisfactorily, ultrathin g-C_3_N_4_ nanosheets with excellent photocatalytic activity and high yield of 25% were obtained successfully at 650 °C for 10 h. A new layer exfoliation and splitting mechanism was also proposed.

## Experimental sections

### Synthesis of g-C_3_N_4_ nanosheets

**Fig. 1:**
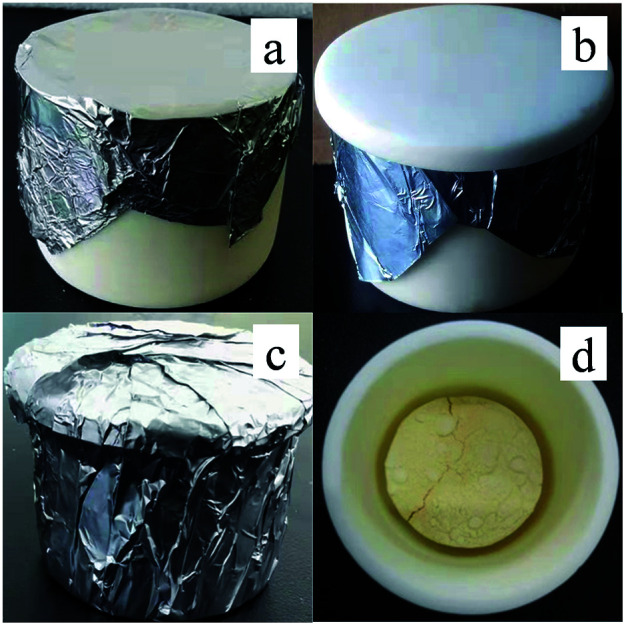
Pictures of the sealed crucible before reaction (a–c) and g-C_3_N_4_ sample S_650-10_ in crucible (d).

The g-C_3_N_4_ nanosheets were synthesized *via* directly heating melamine with a facile template-free one-step synthesis method. A certain mass of melamine was placed in an alumina crucible covered with its alumina lid, and was wrapped with aluminum foil. Then the whole was put in a muffle furnace and heated to a certain temperature (500 °C, 550 °C, 600 °C, 650 °C and 700 °C), and kept for a certain time (4 h, 6 h, 8 h and 10 h) in air, heating ramp is 1 °C min^−1^, followed by cooling down to 200 °C at a cooling rate of 1 °C min^−1^ before natural cooling down to room temperature. The resulting samples were marked as S_600-4_, S_600-6_, S_600-8_, S_600-10_, S_500-10_, S_550-10_, S_650-10_ and S_700-10_, respectively. In addition, in order to further study the effect of sealing system on the yield of sample, we performed the preparation of g-C_3_N_4_ from melamine in an alumina crucible covered with a lid without aluminum foil wrapped at 650 °C for 10 h at ramping rate of 1 °C min^−1^. The sample was marked as S′_650-10_. Fig. 1 shows that pictures of the sealed crucible before reaction and g-C_3_N_4_ sample S_650-10_ in crucible.

### Characterization

The crystal structures of as-prepared samples were identified by power X-ray diffraction (XRD) on a Rigaku Ultima IV X-ray diffractometer at 40 kV and 40 mA with Cu K_α_ radiation (*λ* = 1.5406 Å). The morphologies and structures were determined using environmental scanning electron microscope (ESEM) on an XL-30 ESEM-FEG from FEI Company and transmission electron microscope (TEM) on a FEI TECNAI F20 S-TWIN electron microscope operated at an accelerating voltage of 200 kV. In addition, the morphology and thickness of the nanosheet were characterized by using a Bruker Icon Dimension a tapping-mode atomic force microscopy (AFM) on the mica substrate. Fourier transform infrared (FT-IR) spectra of the samples were obtained on a Nicolet iS5 Fourier transform infrared spectrometer at a resolution of 4 cm^−1^ between 4000 and 400 cm^−1^. The chemical compositions were investigated by using X-ray photoelectron spectroscopy (XPS) on a Thermo ESCALAB 250 instrument with an Al-Kα X-ray radiation. UV-vis diffuse reflection spectra (DRS) were obtained on a TU-1900 Scan UV-vis spectrophotometer equipped with an integrating sphere assembly, using BaSO_4_ as reflectance sample. The photoluminescence spectra (PL) of the samples were surveyed with a F98 fluorescence spectrophotometer made by Shanghai Lengguang Technology Company with an excitation wavelength at 328 nm. Nitrogen adsorption–desorption isotherms were obtained on a ASAP 2020 (V4.01) nitrogen adsorption apparatus from USA, with all samples degassed at 30 °C and a vacuum of 10^−3^ mbar for 6 h before measurements. The thermal stabilities of as-prepared samples were characterized by thermal gravimetric analysis (TGA) by utilizing a Q50 TA thermal analysis instrument from USA under nitrogen gas.

### Evaluation of photocatalytic activity

The photocatalytic activities of the obtained samples were evaluated by degradation of Rhodamine B under visible light irradiation. 15 mg of the photocatalyst was dispersed in a quartz glass tube which contained 30 mL RhB aqueous solution with a concentration of 10 mg L^−1^. Visible light irradiation was provided by a 400 W metal halide lamp and the sodium nitrite solution (1 M) was used to cut off UV light below 400 nm. During irradiation, at selected time intervals, 5 mL of the suspension was taken out and centrifuged at 8000 rpm for 5 min to remove the photocatalyst particles from the solution. The sampled liquid is back to reaction system after each absorbance test, so that the solution in the photo reactor basically remains unchanged. The temporal change of the concentration of RhB was recorded by monitoring the peak value of a maximum absorption of RhB solution by using a TU-1900 UV-vis spectrophotometer. The degradation rate (%) can be calculated according to *η* (%) = [(*C*_0_ − *C*)/*C*_0_] × 100% = [(*A*_0_ − *A*)/*A*_0_] × 100%, where *C*_0_ and *C* respectively are the concentration of RhB solution at the initial moment and *t* moment, and *A*_0_ and *A* respectively represent the corresponding absorbance values.

## Results and discussion

### Yield comparison of samples

The yields of all of samples are listed in Table 1. By comparison of S_600-4_, S_600-6_, S_600-8_, S_600-10_ samples, one can find that the yields of samples are decreased with the prolonging of calcination time. In addition, the yields are also decreased with the increasing of calcination temperature by comparison of S_500-10_, S_550-10_, S_600-10_, S_650-10_ and S_700-10_ samples. This is ascribed to destruction of chemical bond between two tri-*s*-triazine units in polymeric g-C_3_N_4_ at higher temperature, resulting in production of nitrogen and cyano fragments.^42^ The decreased yields can also be confirmed by subsequent TGA analysis. Typically, the g-C_3_N_4_ samples S_600-10_ and S_650-10_ obtained at 600 °C and 650 °C for 10 h have the yield of 40% and 25%, respectively. In addition, the yields of S_700-10_ and S′_650-10_ samples are both zero, although in a tightly sealed alumina crucible system, no any product is obtained at 700 °C. Similarly, no any product is obtained in a semi-sealed alumina crucible system, although at 650 °C.

**Table 1:** Yield comparison of all samples

Sample	S_600-4_	S_600-6_	S_600-8_	S_600-10_	S_500-10_
Yield, %	52	49	45	40	68
Sample	S_550-10_	S_650-10_	S_700-10_	S′_650-10_	
Yield, %	56	25	0	0	

### Morphology and microstructure

**Fig. 2:**
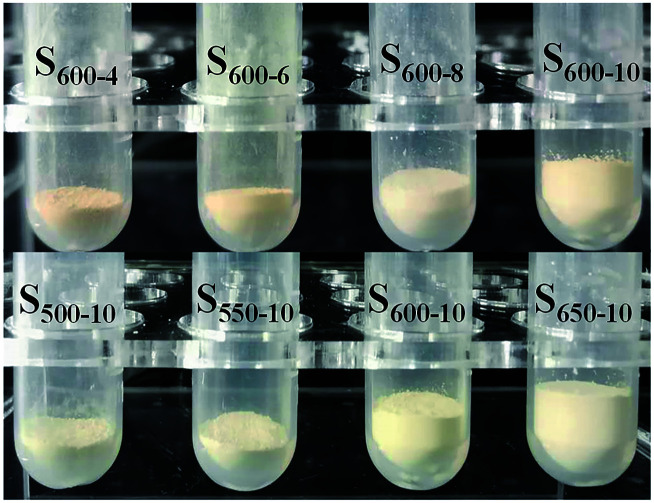
Photograph of volume comparison of samples with the same mass (20 mg).

Fig. 2 shows the photograph of all samples with the same mass of 20 mg, one can clearly see that the volumes of S_600-4_, S_600-6_, S_600-8_ and S_600-10_ samples gradually increase with the prolonging of the calcination time, and those of S_500-10_, S_550-10_, S_600-10_ and S_650-10_ samples also present a tendency to gradually increase with the increasing of the calcination temperature. The volumes of S_600-10_ and S_650-10_ samples are larger than those of other samples, indicating that the g-C_3_N_4_ samples with a loosely stacked state can be obtained at 600 °C and 650 °C for 10 h of the calcination time. Clearly, the volume of S_650-10_ sample is largest, presenting the loosest stacked state.

**Fig. 3:**
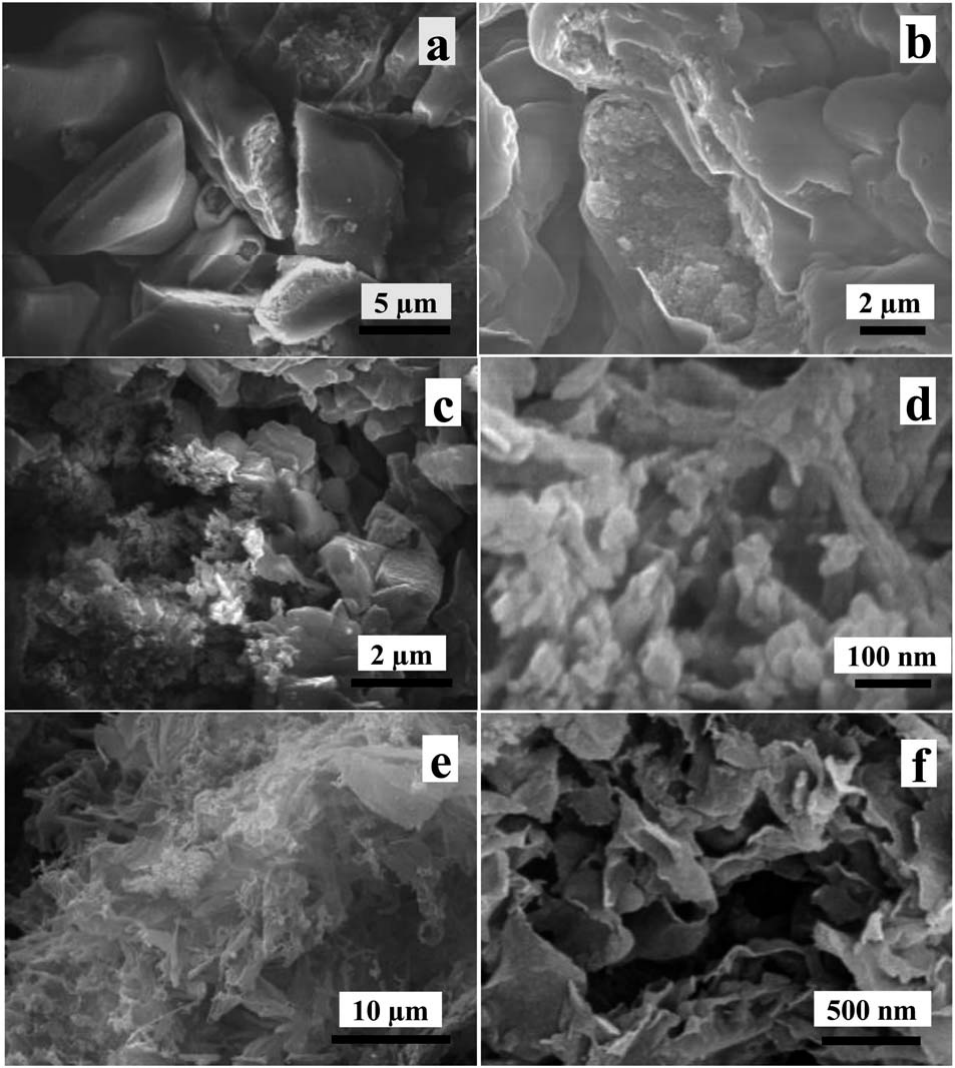
SEM images of S_600-4_ (a), S_600-6_ (b), S_600-8_ (c and d) and S_600-10_ (e) and (f) samples.

The influences of the calcination time and the calcination temperature on the morphology of samples can be further investigated by SEM. SEM images of S_600-4_, S_600-6_, S_600-8_ and S_600-10_ samples calcined at 600 °C for different calcination time are shown in Fig. 3. From Fig. 3a and b, one can see that the two samples exhibit the obvious irregular blocky structure. The surfaces of the blocks are smooth, and the loose inner structure can be observed from some broken blocks. Fig. 3c and d show SEM images with different magnifications of S_600-8_ sample. From Fig. 3c, one can see that the irregular blocks break down, and the inner layers wrapped by the shell get dispersed. The high magnification SEM image of loose inner layers is shown in Fig. 3d, lots of curled sheet structures are observed. SEM images with different magnifications of S_600-10_ sample are shown in Fig. 3e and f. It can be clearly observed, S_600-10_ sample presents curled nanosheet structure. In brief, at the fixed calcination temperature of 600 °C, when the calcination time is extended from 4 h to 10 h, the samples change from bulk structure to loose nanosheet structure with a thickness of several nanometers and a width of several microns. The nanosheet structure is curled because of its high surface energy.

**Fig. 4:**
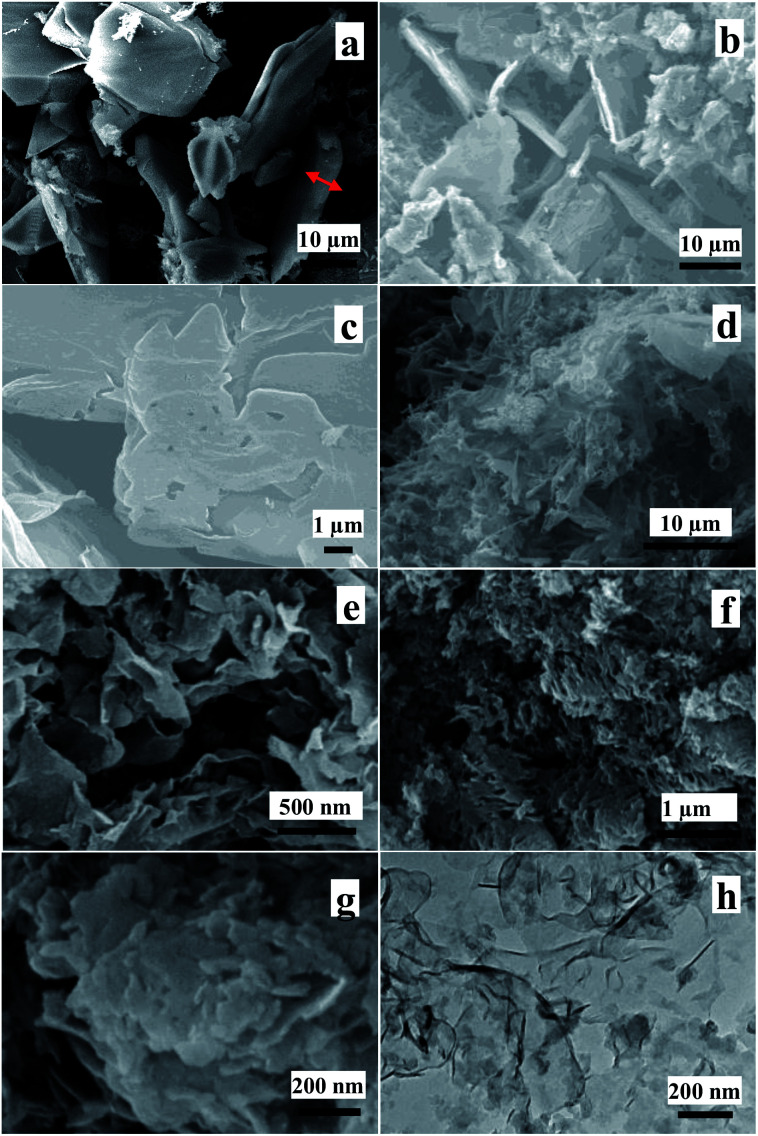
SEM images of S_500-10_ (a), S_550-10_ (b and c), S_600-10_ (d and e) and S_650-10_ (f and g) samples and TEM image of S_650-10_ (h) sample.

Fig. 4 shows that SEM images of S_500-10_, S_550-10_, S_600-10_ and S_650-10_ samples calcined at different calcination temperature for 10 h of calcination time. From Fig. 4a, one can see that S_500-10_ sample exhibits the obvious blocky structure with lateral scale of about 40 μm and thickness of about 2–4 μm. Partial broken structures are observed. SEM image of S_550-10_ sample in Fig. 4b exhibits the layered structure, and the lateral scale and the thickness obviously decrease to about 20 μm and 1 μm, compared with the S_500-10_ sample. In addition, some curled nanosheets are observed. The high magnification SEM image in Fig. 4c further confirms that the multi-layered structure is formed, and the surface has been exfoliated into curved and fragile nanosheets. Fig. 4d and e show SEM images of S_600-10_ sample with different magnification, it can be seen that the multi-layered sheets are almost completely split into ultrathin nanosheets, which show curled state with wrinkles and rolling edges. From Fig. 4e, it can be observed that the lateral scale of nanosheet is several micrometers and the thickness is about several nanometers. Fig. 4f and g show SEM images of S_650-10_ sample with different magnification. It is clear that lots of fragmented structures are observed and the average size of nanosheets is significantly reduced. Fig. 4g shows that the lateral size of most nanosheets is about 100–200 nm. By comparison of all SEM images, one can conclude that S_650-10_ sample has the smallest size, and shows the loosest stack state.

In order to further demonstrate the microstructure of the obtained nanosheets, TEM image of S_650-10_ sample is shown in Fig. 4h. TEM image shows that as-prepared g-C_4_N_4_ has nearly transparent feature, indicating that the g-C_3_N_4_ sample with ultrathin thickness is obtained. Moreover, the curved edges of nanosheets are observed, which is consistent with the analytical results by SEM observation.

**Fig. 5:**
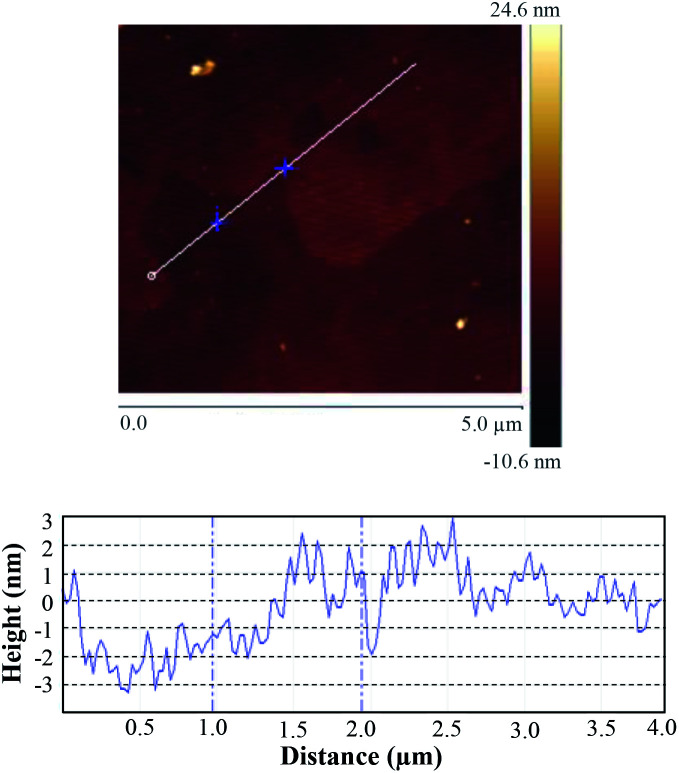
AFM image of S_650-10_ and the corresponding height profile of randomly chosen section.

AFM image and corresponding height profile in Fig. 5 further confirm the ultrathin structure feature of g-C_3_N_4_ nanosheet, and the average thickness is about 2 nm in height, indicating that the multi-layered bulks are successfully exfoliated into ultrathin nanosheets. In addition, the nanosheets with a thickness of more than 3 nm are observed, which is presumably derived from the folds and wrinkles on the nanosheets.

### Crystal structure and chemical compositions

**Fig. 6:**
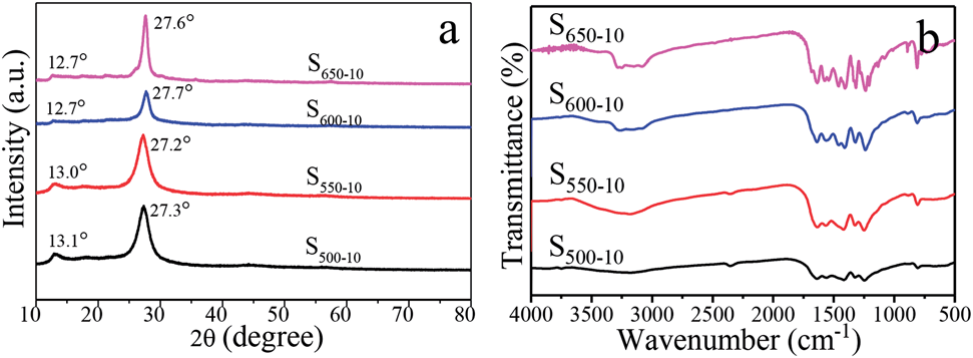
XRD patterns (a) and FT-IR spectra (b) of S_500-10_, S_550-10_, S_600-10_ and S_650-10_ samples.

The XRD patterns of S_500-10_, S_550-10_, S_600-10_ and S_650-10_ samples obtained from different calcination temperature are shown in Fig. 6a. The XRD patterns of all of samples exhibit similar characteristic diffraction peaks at around 13.0° and 27.6°, which are consistent with the typical graphite-like hexagonal phase of g-C_3_N_4_ (JCPDS 87-1526). The low-angle diffraction peak around 13.0° assigned to (100) plane corresponds to the in-plane structural stacking of tri-*s*-triazine units, the typical dominant peak around 27.6° assigned to (002) plane is attributed to the inter-layer stacking of the conjugated aromatic systems, corresponding to the interplanar distances of 0.326 nm and 0.675 nm, respectively.^43,44^ In addition, one can see that the diffraction angle 2*θ* of (002) peak increases from 27.3° and 27.2° for S_500-10_ and S_550-10_ samples to 27.7° and 27.6° for S_600-10_ and S_650-10_ samples when the calcination temperature increases from 500 °C to 650 °C. This result may be attributed to the stack of g-C_3_N_4_ becomes denser and more ordered at higher calcination temperature.^31^ More interestingly, the intensity of diffraction peak decreases when the calcination temperature increases from 500 °C to 600 °C, while the intensity significantly increases when the temperature increases to 650 °C. The decrease in peak intensity of S_600-10_ sample may be interpreted as the more structural defects in sample. The increase in peak intensity of S_650-10_ sample could be attributed to the reduced number of structural defects and the enhanced crystallinity due to the formation of perfect tri-*s*-triazine (melem) structure units when the calcination temperature increases to 650 °C.^31,37^

The surface functional groups of the obtained g-C_3_N_4_ samples are monitored by FT-IR spectroscopy, and the spectra of three samples are shown in Fig. 6b. The strong absorption bands further reveal a typical graphitic carbon nitride molecular structure feature. The broad absorption band in the region from 3000 cm^−1^ to 3500 cm^−1^ originates from the uncondensed terminal amino groups (–NH_2_ or =NH).^28^ Several strong bands in the 1230–1650 cm^−1^ region are attributed to the stretching vibrations of C–N and C=N from aromatic heterocycles.^28^ The sharp peak at around 805 cm^−1^ is assigned to the bending vibration of triazine rings.^28^ By comparison, it can be seen that the absorption peaks of S_650-10_ became sharper than those of the other samples, which may be interpreted as the rearrangement of the CN units and the more ordered packing of the polymeric tri-*s*-triazine (melem) units at higher calcination temperature.^31,37^

**Fig. 7:**
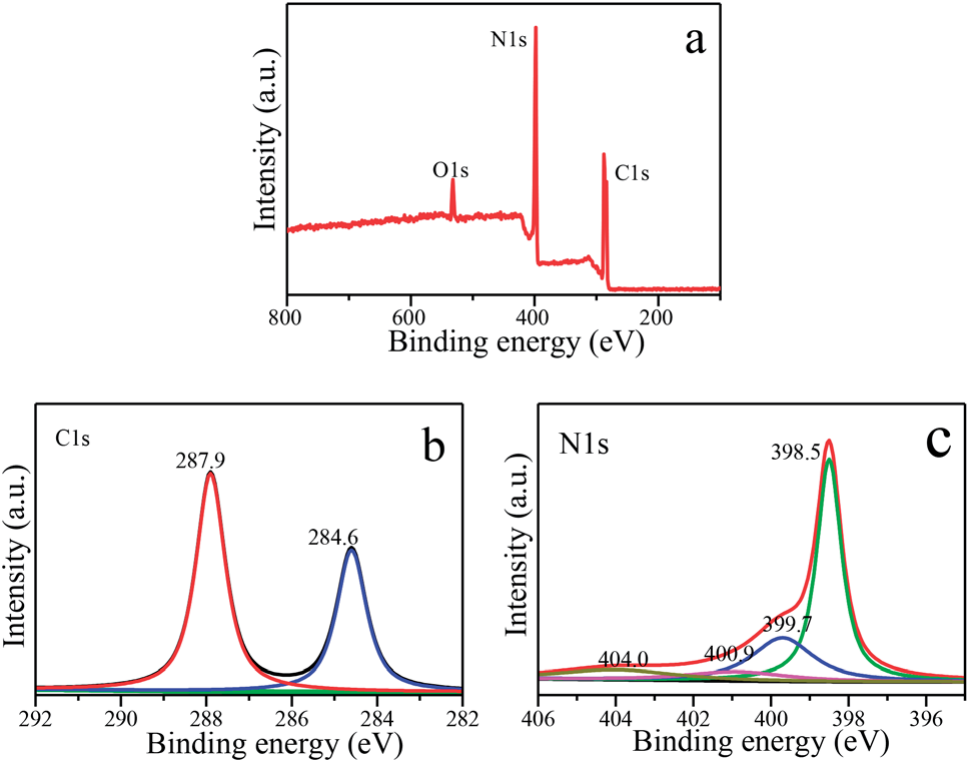
XPS spectra of S_650-10_ sample. (a) XPS survey. (b) C_1s_ spectra. (c) N_1s_ spectra.

The obtained S_650-10_ nanosheet as a representative example is further studied by XPS to reveal its chemical composition and oxidation state, as shown in Fig. 7a–c. It is clear that C, N and O elements are detected in the XPS survey spectra in Fig. 7a. The O_1s_ peak may be derived from the surface absorbed oxygen species. The backbone C and N elements of S_650-10_ are further investigated by the high resolution spectra. For C_1s_ spectra in Fig. 7b, a predominant peak at 287.9 eV is observed in nanosheet, which is assigned to the N=C–(N)_2_ bonds in the g-C_3_N_4_ lattice.^6^ The peak at 284.6 eV is assigned to C–C bonds, which is related to adventitious carbon species.^45^ The high resolution N_1s_ spectra in Fig. 7c show four different peaks at 398.5, 399.7, 400.9, 404.0 eV, which can be ascribed to the sp^2^-hybridized nitrogen in triazine rings (C=N–C), the tertiary nitrogen groups (N–(C)_3_), the free amino groups ((C)_2_N–H or C–NH_2_) and π-excitations, respectively.^6^

### BET surface areas and pore size distribution

**Fig. 8:**
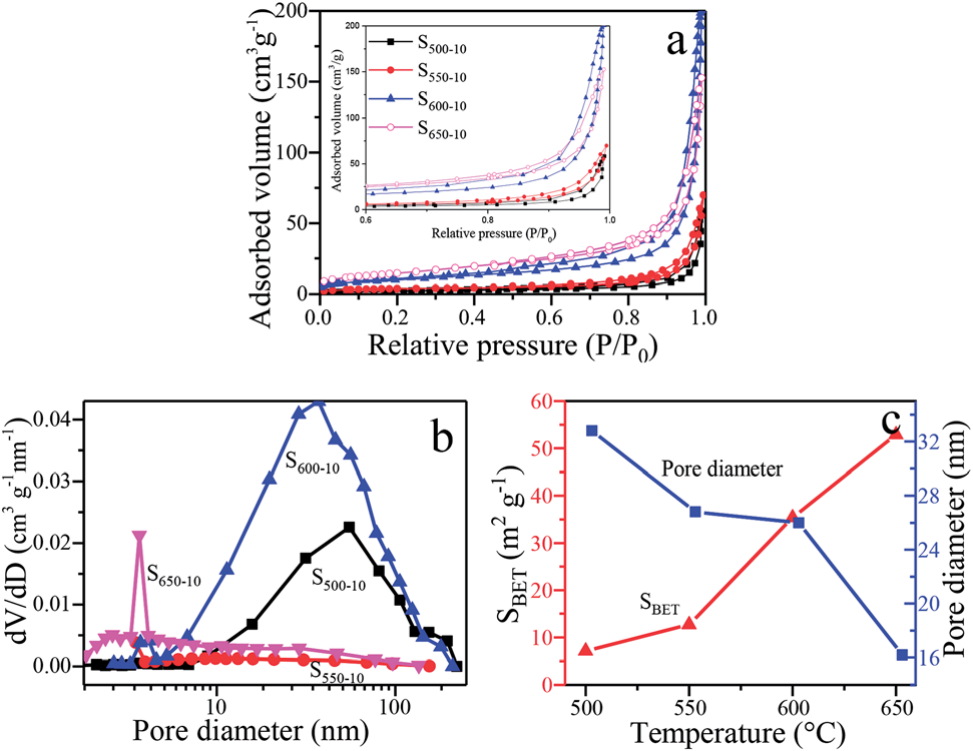
(a) N_2_ adsorption–desorption isotherms, the inset is a magnification when the relative pressure is 0.6–1.0. (b) The corresponding PSD curves and (c) the correlation between S_BET_, APD and temperature of S_500-10_, S_550-10_, S_600-10_, S_650-10_ samples.

In order to characterize the specific surface areas and porosity of g-C_3_N_4_ samples obtained under different calcination temperature, the nitrogen adsorption–desorption isotherms and Barrett–Joyner–Halenda (BJH) pore size distributions (PSD) are shown in Fig. 8. The illustration is a magnification when the relative pressure is 0.6–1.0. From Fig. 8a, the type IV (BDDT classification) shape of adsorption–desorption isotherms with an H3-type hysteresis loops for all the samples can be observed, suggesting the presence of slit-shaped mesopores derived from the aggregates of sheet-like particles.^31,37^ The correlation of the BET surface areas, the BJH desorption average pore diameter and the calcination temperature for S_500-10_, S_550-10_, S_600-10_, S_650-10_ samples is indicated in Fig. 8c. It can be clearly seen that the surface areas of the samples are remarkably increased and average pore diameter decreased with the increase of calcination temperature. The BET surface areas (S_BET_) of S_500-10_, S_550-10_, S_600-10_, S_650-10_ samples are respectively 7.1 m^2^ g^−1^, 12.7 m^2^ g^−1^, 35.4 m^2^ g^−1^ and 52.9 m^2^ g^−1^, and the BJH desorption average pore diameter (APD) are respectively 33, 27, 26, 16 nm (Table 2). From Fig. 8b, the PSD of S_500-10_ sample ranges from 10 nm to 110 nm, the larger mesopores are related to the pores formed between packed layers. Most probable aperture is 55 nm. With regard to S_550-10_ sample, the pore size distribution is from 7 nm to 30 nm, and most probable aperture is 10 nm. In addition, small mesopores with the diameter less than 3.5 nm are observed. The pore area is obviously reduced compared with S_500-10_ sample, indicating that the multilayer structure has been exfoliated to monolayer or a few layers structure. This is consistent with SEM images of S_500-10_ and S_550-10_ samples (Fig. 4a and b). For S_600-10_ sample, the pore area is obviously increased compared with other samples, the PSD is from 10 nm to 110 nm, and most probable aperture is 37 nm. The larger mesopores are related to the pores formed from curled sheets (Fig. 4d). For S_650-10_ sample, the PSD curve is quite broad (from 1 nm to 100 nm) with small mesopores and large mesopores, and most probable aperture is 4 nm. The proportion of small mesopores is more larger than large mesopores, and the smaller mesopores may reflect porosity within the nanoscale sheets. This is because that S_650-10_ sample presents small and thin nanosheet structure and has best dispersion due to the higher calcination temperature of 650 °C. This has been confirmed in SEM images in Fig. 4f and g. The analysis results indicate that the specific surface area of samples increase with the increase of the calcination temperature, which can be attributed to the decreased size and thickness of g-C_3_N_4_ samples. Generally, the g-C_3_N_4_ nanosheets with mesoporosity have enlarged specific surface area. The enlarged specific surface area could improve mass transfer ability and provide larger number of active redox reaction sites, and further efficiently enhance photocatalytic activity.^38^

**Table 2:** *S*_BET_ and APD of samples

Sample	S_500-10_	S_550-10_	S_600-10_	S_650-10_
*S*_BET_ (m^2^ g^−1^)	7.1	12.7	35.4	52.9
APD (nm)	33	27	26	16

### Optical property and band gap

**Fig. 9:**
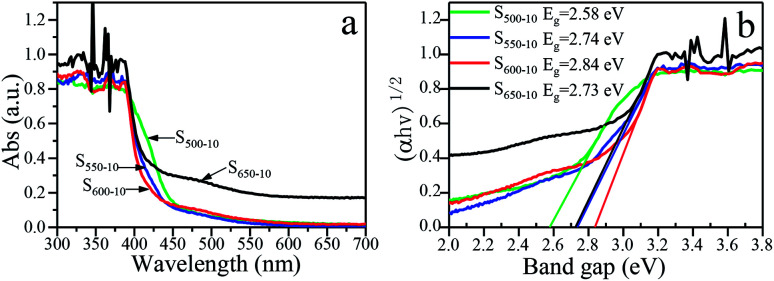
(a) UV-vis DRS spectra and (b) plots of (*αhν*)^1/2^*vs.* photon energy of S_500-10_, S_550-10_, S_600-10_ and S_650-10_ samples.

The effects of the calcination temperature on the optical property are investigated by UV-vis DRS spectra and PL spectra. The photoabsorption abilities of S_500-10_, S_550-10_, S_600-10_ and S_650-10_ samples are characterized with UV-vis absorption spectrum, as shown in Fig. 9a. Obviously, all the g-C_3_N_4_ samples exhibit an absorption edge in the visible light region. It is worth noting that S_550-10_, S_600-10_ and S_650-10_ samples show clear hypsochromic shifts on the absorption edge compared with S_500-10_ sample. In addition, the UV-visible absorption spectra of S_650-10_ sample shows a significant enhancement of the absorption in the visible region compared with the other samples. Besides, the absorption in the UV region is also enhanced. The calculated band gap energy values (*E*_g_) of g-C_3_N_4_ samples on the basis of the UV-vis DRS data are indicated in Fig. 9b, it can be seen that band gap energy is increased from 2.58 eV to 2.84 eV when the calcination temperature increases from 500 °C to 600 °C, then is slightly decreased from 2.84 eV to 2.73 eV when the calcination temperature increases from 600 °C to 650 °C.

The hypsochromic shift on the absorption edge of the UV-visible absorption spectra can be ascribed to the quantum confinement effect induced by nanosized particles. As is shown in Fig. 4, the higher temperature makes the thickness and size of g-C_3_N_4_ layers evidently decreased, resulting in the evident quantum confinement effect. Because of the quantum confinement effect, the energy levels on the conduction band and valence band become separated levels, the energy gap gets widen, the valence band potential become more positive and the conduction band potential become more negative, which increases the redox ability of photogenerated holes and electrons. This will further enhance the photocatalytic activity of the samples.

For S_650-10_ sample, the slight decrease of band gap energy can be explained by the unique effect of the calcination temperature on the molecular structure and crystal structure of the polymeric g-C_3_N_4_. At the calcination temperature of 650 °C, the obtained g-C_3_N_4_ nanosheets should be mainly constructed from tri-*s*-triazine (melem) structure units, the structure units show enhanced structural connections, which would result in the decrease of band gap of g-C_3_N_4_.^37^ In addition, the enhancement of the absorption in the visible region and UV region for S_650-10_ sample may be ascribed to the larger specific surface area, which can absorb more light energy to generate more electron hole pairs. Photogenerated carriers can easily migrate from the interior of particles to the surface through simple diffusion, and take part in the redox reaction with electron donor or acceptor. The less time the electrons diffuse from the interior to the surface, the higher the separation efficiency of the photogenerated charge is. This higher separation efficiency could improve the probability of their involvement in photocatalytic reaction before recombination and enhance the photocatalytic activity.

**Fig. 10:**
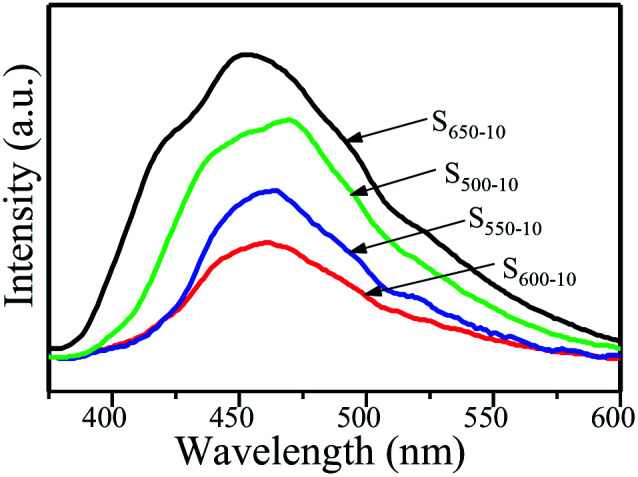
Room-temperature PL spectra of S_500-10_, S_550-10_, S_600-10_ and S_650-10_ samples under 328 nm excitation.

Commonly, the separation and recombination of the photogenerated charge-carriers are monitored by photoluminescence (PL) spectra. The PL spectra of S_500-10_, S_550-10_, S_600-10_ and S_650-10_ samples with an excitation wavelength of 328 nm are shown in Fig. 10. All of samples exhibit the obvious PL emission peaks centered at about 450–470 nm, indicating that the π-conjugated system of C_3_N_4_ samples is constant under different calcination temperature. Interestingly, for S_500-10_, S_550-10_ and S_600-10_ samples, a drastic quenching phenomenon of the PL peak intensity is observed with the increase of the calcination temperature, especially for S_600-10_ sample, but PL peak intensity of S_650-10_ sample is remarkably enhanced. For S_600-10_ sample, the decline of PL intensity should be attributed to the more structural defects in the samples and decreased crystallinity. When the temperature is at 600 °C, multi-layered sheet-like g-C_3_N_4_ sample are almost completely split into ultrathin nanosheets, the specific surface areas significantly increase, which may result in more defects and poor crystallinity. More structural defects in the samples could capture the electrons or holes and prevent the recombination probability of photogenerated electron–hole pairs and thus lead to the decline in PL intensity.^31,37^ For S_650-10_ sample, the enhanced PL intensity should be attributed to the improved crystal structure and the reduced number of structural defects. When the temperature increases to 650 °C, the crystallinity of S_650-10_ sample is improved due to the formation of perfect tri-*s*-triazine (melem) structure units with enhanced structural connections, and the number of structural defects is reduced, the recombination probability of photogenerated electron–hole pairs is increased and thus the PL intensity is enhanced.^31,37^ Although the recombination probability of photogenerated electron–hole pairs is reduced, the transitions of electrons or holes to the defects belongs to harmful radiationless transitions, which is equivalent to reducing the effective utilization ratio of electrons or holes participating in the redox reactions of the organic pollutants. Therefore, both the PL intensity and photocatalytic activity of S_650-10_ sample are significantly enhanced by decreasing harmful radiationless transitions.

### Thermal stability

**Fig. 11:**
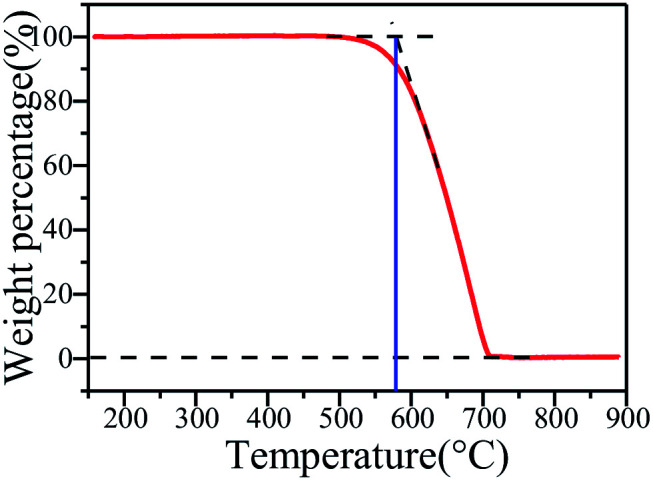
TGA curve of g-C_3_N_4_ sample S_650-10_.

The thermal stability of g-C_3_N_4_ sample (S_650-10_) is characterized by TGA. The TGA curve of g-C_3_N_4_ is illustrated in Fig. 11. It can be seen that the TGA curve displays that its decomposition temperature begins at about 580 °C, and the weight loss reaches 100% at about 700 °C.^46^ This is ascribed to destruction of chemical bond between two tri-*s*-triazine units in polymeric g-C_3_N_4_ at high temperature, resulting in production of nitrogen and cyano fragments.^42^

### Formation mechanism

**Scheme 1:**
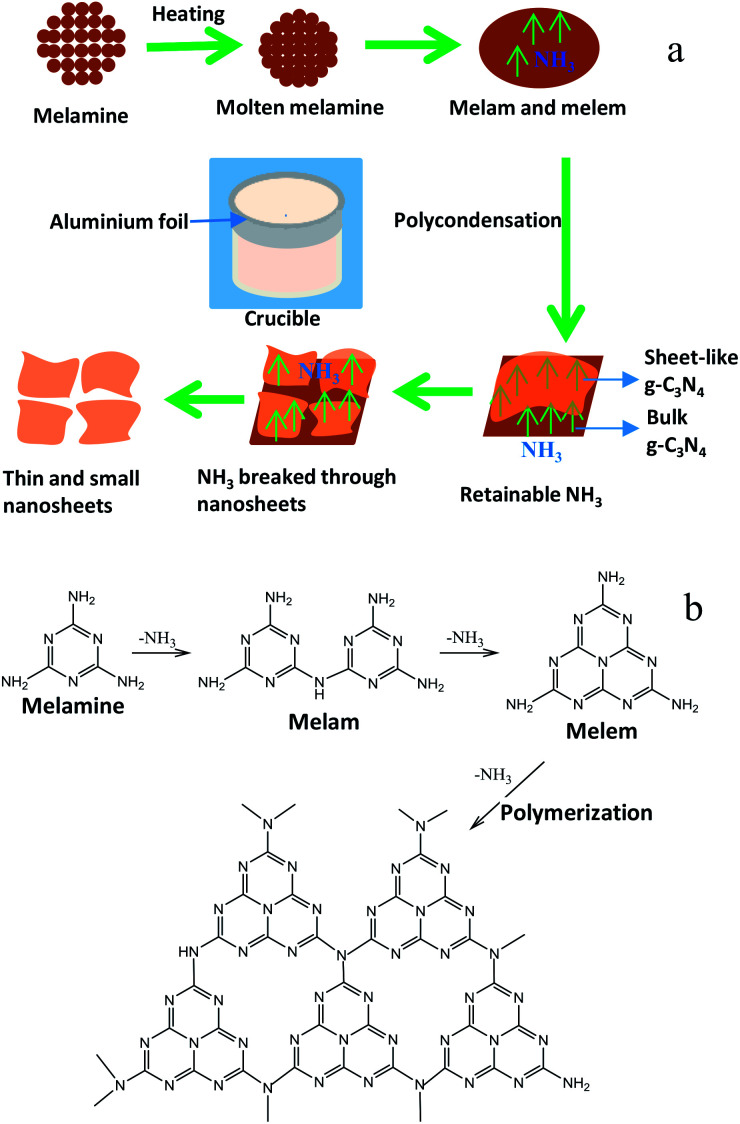
Schematic for the formation (a) and the reaction paths (b) of g-C_3_N_4_ nanosheet from the melamine precursor.

A new layer exfoliation and splitting mechanism of the formation of the ultrathin nanosheet is proposed. In the present work, a certain mass of melamine powders are placed in an alumina crucible covered with its lid and tightly wrapped by aluminum foil, then the whole is put in a muffle furnace and heated to 650 °C at a heating rate of 1 °C min^−1^, and kept for 10 h at 650 °C. Schematic for the formation mechanism of g-C_3_N_4_ nanosheet from the melamine precursor is shown in Scheme 1a. When the temperature of system rises to above 250 °C, melamine powders turn molten, and the melamine molecules begin to gather together and the molecular spacing become smaller. As temperature rises gradually, the intermolecular condensation reaction of melamine occurs, and small amounts of ammonia is generated. Melam and melem are main condensation products at an initial stage. When the temperature increases to about 450 °C, the thermal polycondensation reaction among melam, melem and melamine oligomer molecules begins to occur, and the tris-*s*-triazine structure begin to form preliminarily. At this time, the reaction rate is slow and the amount of ammonia released is less. In order to increase the reaction rate, the temperature is raised to 650 °C. As is known to all, the polycondensation reaction is a step-by-step procedure, so that the formation of polymeric g-C_3_N_4_ needs a longer time to achieve a high degree of polycondensation. In view of this, the thermal treatment time is prolonged to 10 h. The reaction system is maintained at 650 °C for 10 h. With the increases of the degree of polycondensation, the generated ammonia is constantly increased. Due to the reaction system is tightly sealed, ammonia gas can keep circulating in system at a longer time. The circulating flow of large amounts of ammonia between layers of g-C_3_N_4_ favors not only the direct formation of ultrathin nanosheet structure bypass the transition state of bulk structure, but also the exfoliation of layered bulk structure into nanosheets. Finally, the favourable nanosheets are formed successfully, and some large size nanosheets are split into small size nanosheets. The result suggests that the thinner and looser nanoarchitectures of g-C_3_N_4_ can be obtained by prolonging the heat treatment time and increasing heat treatment temperature. This is consistent with the reported results in literatures.^37,47,48^ In conclusion, the retainable self-supporting ammonia, and the high calcination temperature and the prolonged calcination time co-contribute to the formation of ultrathin g-C_3_N_4_ nanosheets. Additionally, chemical reaction paths from the polycondensation of melamine precursor into g-C_3_N_4_ are shown in Scheme 1b.^49^

### Photocatalytic activity and cycling stability

**Fig. 12:**
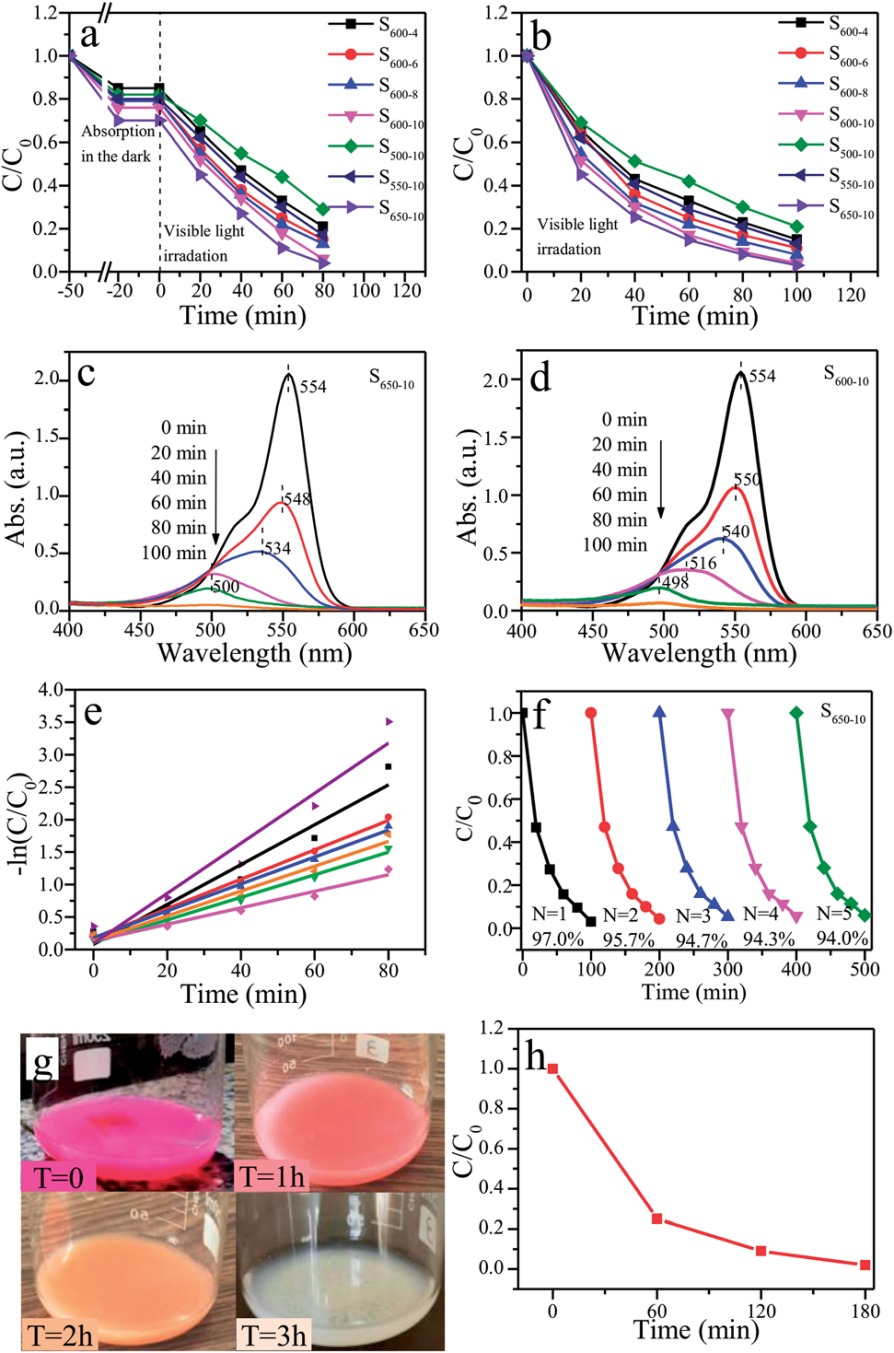
The time-dependent curves of RhB concentration change under visible light irradiation (*λ* > 400 nm) ((a) pre-absorption, (b) without pre-absorption). The temporal absorption spectra of RhB in the presence of S_650-10_ (c) and S_600-10_ (d) samples. (e) Linear transform −ln (C/C_0_) of the kinetic curves of RhB degradation. (f) Photocatalytic reaction cycles of S_650-10_ sample. (g) The color change pictures and (h) concentration change curve of RhB solution over S_650-10_ sample with time under natural sunlight.

The photocatalytic activities of all prepared g-C_3_N_4_ samples (S_600-4_, S_600-6_, S_600-8_, S_600-10_, S_500-10_, S_550-10_, S_600-10_ and S_650-10_) are evaluated by degradating RhB aqueous solution under visible light irradiation. Two methods are used in photocatalytic experiments, one is that the catalyst samples and RhB solution mixture are magnetically stirred for 30 minutes in a dark before visible light irradiation for pre-adsorption, the other is that the mixture are directly used in photocatalytic experiments without pre-adsorption. Fig. 12a and b show the time-dependent curves of RhB concentration change in two cases. It can be seen from Fig. 12a, after being stirred for 30 min in the dark, the concentrations of RhB solutions decreased by 15%–30%, which indicating that the prepared samples have strong adsorption capacity on RhB. At this time, adsorption–desorption equilibrium is achieved in the mixed system. Under visible-light irradiation, the concentrations of RhB solutions in the presence of g-C_3_N_4_ samples show notable downward trends. This indicates that all prepared samples have good photocatalytic activities over the degradation of RhB. By comparison of the concentration change curves of RhB in the presence of S_600-4_, S_600-6_, S_600-8_ and S_600-10_ samples, one can see that S_600-10_ sample presents the highest photocatalytic activity. The corresponding degradation rate of RhB reaches 94% after 80 min irradiation. This indicates that the photocatalytic activities of samples will be improved with the prolonging of the calcination time when the calcination temperature is constant. Compared with S_500-10_, S_550-10_ and S_600-10_ samples, S_650-10_ sample show the highest photocatalytic activity, and the degradation rate of RhB is 96%. This implies that the photocatalytic activities of samples will be enhanced with the increase of the calcination temperature when the calcination time is constant. Moreover, among all the samples, S_650-10_ sample shows highest photocatalytic degradation efficiency over RhB. From Fig. 12b, it can be seen that the concentration change rate of RhB during the first 20 min visible-light irradiation are the largest. With the increase of irradiation time, the decline rates of RhB concentration slow down gradually. The primary reason is that, the concentration of RhB without pre-absorption are greater in the early stage of irradiation, and strong adsorption and photocatalysis proceed simultaneously so that the degradation rate of RhB is faster. Obviously, S_600-10_ and S_650-10_ samples show highest photocatalytic activities over RhB, which is consistent with the results from Fig. 12a. The corresponding degradation rate are respectively 96% and 97% after 100 min irradiation. The photocatalytic activity of S_650-10_ sample is better than that of S_600-10_ sample. This is because that S_650-10_ sample has stronger adsorption capacity, which is related to the larger specific surface area.

Dong *et al.* reported that RhB degradation ratio of g-C_3_N_4_ samples is 100% after 300 min irradiation (RhB: 5 mg L^−1^, g-C_3_N_4_: 0.9 g L^−1^, 500 W Xe lamp).^37^ However, in our present work, RhB degradation ratio of g-C_3_N_4_ samples is almost 100% after 100 min irradiation (RhB: 10 mg L^−1^, g-C_3_N_4_: 0.5 g L^−1^, 400 W Metal halide lamp). At present, due to no unified photocatalytic experiment standard, it is difficult to keep the same test conditions for different researchers. The factors of pollutant concentration, catalyst dosage and the types and power of radiation sources *etc.* have great influence on photocatalytic degradation rate. It is generally believed that the higher contaminants concentration and catalyst dosage are, the better the degradation efficiency of contaminants is. Therefore, it can be concluded that the g-C_3_N_4_ samples prepared by our group have better photocatalytic performance.

Besides, the temporal absorption spectra changes of RhB in the presence of S_650-10_ and S_600-10_ samples are shown in Fig. 12c and d. The declines of absorbances are mainly attributed to the degradation reaction of RhB catalyzed by S_650-10_ and S_600-10_ samples. These observations indicate that the whole conjugated chromophore structure of RhB undergo a facile cleavage. Notably, the concomitant slight hypsochromic shifts are observed, indicating that a de-ethylation process simultaneously occurrs.^29^ It can be seen from Fig. 12c, after 60 min irradiation, the maximum absorption wavelength shifts from 554 nm to 500 nm. Moreover, in the course of experiment, we find the colour of the RhB solution turns to colourless after 60 min irradiation. This can be confirmed by the absorbance value at 554 nm in absorption spectrum curve. From Fig. 12d, we can find that the maximum absorption wavelength shifts to 516 nm after 60 min irradiation, and shifts to 498 nm after 80 min irradiation. It indicates that the degradation rate of RhB is different under the action of different catalyst samples. By the comparison of two absorption spectra, we can also conclude that S_650-10_ sample possesses more excellent photocatalytic activity than S_600-10_ sample.

The kinetic degradation curves of RhB in the presence of various samples are further analysed, as shown in Fig. 12e. The degradation rate constants of RhB are respectively 0.01272 min^−1^ (S_500-10_), 0.01737 min^−1^ (S_600-4_), 0.01912 min^−1^ (S_550-10_), 0.02073 min^−1^ (S_600-6_), 0.02263 min^−1^ (S_600-8_), 0.03069 min^−1^ (S_600-10_) and 0.03854 min^−1^ (S_650-10_) (Table 3). S_650-10_ sample possesses the highest photocatalytic degradation ability on RhB, and the degradation rate constant of RhB reaches 0.03854 min^−1^. However, the highest degradation rate constant of RhB over g-C_3_N_4_ reported by Dong *et al.* is only 0.0165 min^−1^.^37^ These kinetics degradation curves basically conform to the first-order reaction kinetics process. In the later stage of degradation reaction, the phenomenon of deviation from first-order reaction kinetics occurs because of very low concentration of RhB.

**Table 3:** Rate constants of samples

Samples	S_500-10_	S_600-4_	S_550-10_	S_600-6_
Rate constants/min^−1^	0.01272	0.01737	0.01912	0.02073
Samples	S_600-8_	S_600-10_	S_650-10_	
Rate constants/min^−1^	0.02263	0.03069	0.03854	

The assessment of photochemical stability of photocatalysts is important for practical applications. The reusability tests over S_650-10_ sample are performed by carrying out five consecutive runs for the degradation of RhB under identical reaction conditions. The used samples are recycled by centrifugation, washed with ethanol and water for several times, and then dried at 60 °C for 2 h. Five photocatalytic reaction runs of the S_650-10_ for RhB are shown in Fig. 12f. One can find that the degradation rate of RhB only decreases very slightly after five runs, indicating that S_650-10_ sample possesses excellent stability. Consequently, the as-prepared g-C_3_N_4_ is a promising visible light photocatalyst in the fields of air purification and wastewater treatment.

In addition, in order to provide usefulness for catalysts in the practical application, the photocatalytic experiment of S_650-10_ sample under natural sunlight is performed on the indoor windowsill (date: 5 October 2019, real time temperature of test location: 23–25 °C, longitude: 125.37, latitude: 43.87). The color change process of RhB over S_650-10_ sample under natural sunlight is recorded by digital photos and is shown in Fig. 12g. It can be clearly seen that the color of RhB turns from pink to colourless after 180 min under natural sunlight irradiation. The photocatalytic degradation curve of RhB is indicated in Fig. 12f. The degradation rate of RhB is almost 100% after 180 min. Hence, the prepared S_650-10_ sample has practical application value.

The remarkably enhanced photocatalytic activities for S_650-10_ sample can be interpreted as the synergistic effects of the enhanced crystallinity, the large surface area, the reduced layer thickness and size, and the reduced number of defects. Firstly, the higher heat treatment temperature and the longer heat treatment time make the layer thickness and size of the as-prepared g-C_3_N_4_ reduced significantly, resulting in the evident quantum confinement effect. The quantum confinement effect makes the energy gap get widen, and the valence band potential become more positive and the conduction band potential become more negative, which effectively improve the redox ability of photogenerated holes and electrons. Moreover, the transport ability of photogenerated carriers in nanosheet is also enhanced and the separation efficiency of electron–hole pairs is improved. This higher separation efficiency could improve the probability of their involvement in photocatalytic reaction before recombination and enhance the photocatalytic activity. Secondly, the enlarged specific surface area could improve mass transfer ability and provide larger number of active redox reaction sites, which contributes to adsorbing abundant reactant molecules and promotes interfacial photocatalytic redox reactions with substrates.^29^ Finally, the higher crystallinity is advantageous to reduce the number of the defects.^37^ These favorable properties co-contribute to the improvement of photocatalytic activities of g-C_3_N_4_ nanosheets under visible light irradiation.

## Conclusions

In summary, a facile template-free one-step synthesis method of ultrathin g-C_3_N_4_ nanosheets is developed though thermal polycondensation of melamine at 650 °C for 10 h. The ultrathin g-C_3_N_4_ nanosheets with high yield of 25% are obtained at 650 °C for 10 h. The formation of ultrathin g-C_3_N_4_ nanosheets depends mainly on the higher heat treatment temperature, the longer heat treatment time and the retainable self-supporting ammonia generated from polycondensation reaction due to tightly sealed reaction system and low heating rate. The enhanced crystallinity, the large surface area, the reduced layer thickness and size, and the reduced number of defects co-contribute to the enhanced visible light photocatalytic activities of g-C_3_N_4_ nanosheets. A new layer exfoliation and splitting mechanism of formation of the ultrathin nanosheets is proposed. The visible light photocatalytic activity of g-C_3_N_4_ nanosheets over RhB degradation are significantly improved as the heat treatment temperature is increased and the heat treatment time is prolonged. Moreover, g-C_3_N_4_ nanosheets have excellent photochemical stability. This work provides a new strategy to develop a facile eco-friendly template-free one-step synthesis method for potential large-scale synthesis of g-C_3_N_4_ nanosheets with high yield, high efficiency and stable activity for environmental and energetic applications.

## Conflicts of interest

There are no conflicts of interest to declare.

## Supplementary Material
